# Secondary hypertension for the generalist: When do you refer to hypertension specialist clinics, and how do you screen for secondary hypertension?

**DOI:** 10.1016/j.clinme.2026.100597

**Published:** 2026-05-22

**Authors:** Christopher E. Clark, Anish George

**Affiliations:** a Department of Health and Community Sciences, University of Exeter, Smeall Building, St Luke's Campus, Magdalen Rd, Exeter EX1 2LU, United Kingdom; b Royal Devon University Hospital, Barrack Road, Exeter, Devon EX2 5DW, United Kingdom

**Keywords:** Hypertension

## Abstract

Current guidelines recommend referral to adult hypertension specialists when further investigation is likely to influence management. This article focuses on identifying aspects of adult hypertension that should lead to specialist referral and/or investigation, being relevant to both primary and secondary care.

## Highlights

• Hypertension affects one in three adults worldwide. This prevalence means that people with hypertension are encountered in all clinical specialties, so a working knowledge of hypertension is essential for all clinicians.
• Blood pressure measurement is a skill that should be carefully conducted according to guidance, to avoid erroneous readings and management.
• Symptoms and signs of hypertension are strong indicators of uncontrolled blood pressure, so should be considered whenever high blood pressure is detected.
• Signs of target organ damage out of proportion to measured blood pressures suggest possible secondary causes of hypertension, and indicate a need for further investigation.
• High blood pressure in pregnancy can have serious sequelae and requires careful collaborative care across specialties whenever detected.

## Introduction

Hypertension is the most prevalent modifiable cardiovascular risk factor and is the leading cause of morbidity and mortality worldwide.^1^ Most adults with hypertension (90%) are diagnosed and managed entirely in primary care, using lifestyle interventions and guideline-directed pharmacotherapy.^2^ Given its high prevalence, hypertension may also be encountered, or first recognised, in outpatient, inpatient or emergency settings, either incidentally or in relation to the presenting complaint. A working knowledge of the referral and investigation pathways is, therefore, essential across healthcare settings.

## Things to consider before referring

Most secondary care hypertension clinics in the UK are oversubscribed. It is, therefore, worth considering measures to optimise blood pressure (BP) control prior to referral and identifying patients most likely to benefit from specialist assessment.

In every setting, care should be taken to ensure accurate BP measurements, considering the posture, technique, rest periods, environment and equipment (Tables 1 and 2).^3^ Clinical staff should be trained, and regularly refreshed, in accurate BP measurement. This will avoid over- or underestimation of BP, minimise white-coat effects and avoid erroneous referrals.^4^, ^5^ At initial assessment, BP should be measured in both arms, and the higher arm value recorded for diagnosis and treatment.^6^ Diagnosis of hypertension should be confirmed using ambulatory (ABPM) or home BP monitoring before referral, unless an urgent referral is clinically indicated (see below).^4^ Once hypertension is confirmed, and following lifestyle interventions, pharmacological therapy should be initiated in accordance with the National Institute for Health and Care Excellence (NICE) guideline NG136.^4^

**Table 1:** Factors affecting the accuracy of BP measurement.

Factor	Magnitude of systolic/diastolic BP discrepancy (mmHg)
Talking or active listening	1+4–19/+5–13
Distended bladder	+4–33/+2–18
Smoking within 30 min of measurement	+2–25/+2–18
Caffeine within 30 min of measurement	+3–14/+2–13
Arm unsupported, sitting	+4.9/+3–5
Arm unsupported, standing	+1–7/+5–11
Back unsupported	Nil significant/+6.5
Acute meal ingestion	−6/−5 to −2

**Table 2:** Considerations for accurate brachial blood pressure measurement.

Steps	Considerations
Preparation	Avoid caffeine, alcohol and smoking for 30 min beforehand Rest seated in a quiet area for 5 min before measurement
Positioning	Sit upright with back supported Support upper arm at heart level^a^ Support forearm in order to relax shoulder and upper arm Legs should be uncrossed and feet flat on the floor
Affix the cuff	Select the correct size cuff for arm circumference, typically Standard = 22–32 cm Large = 32–42 cm Extra-large = 42–50 cm^b^ Position cuff 1–2 cm above elbow flexure, centring bladder on brachial artery (note any artery marker to guide positioning) Apply to bare arm, not over clothing
Taking measurements	Maintain recommended posture throughout Do not talk If BP ≥140/90, take a second measurement If second measurement substantially differs, take a third measurement Measure both arms, in case of difference
Recording BP	From the higher-reading arm, record the lower of the second or third measurements, unless only a single measurement made Record which is the higher-reading arm, for future reference Record lower-reading arm BP, noting inter-arm BP difference

a For wrist devices, same sitting posture, support wrist at heart level, position palm according to device manufacturer instructions.

b Quoted cuff ranges vary by manufacturer, some being wide ranging, typically 22–42 cm. Paediatric cuffs should also be available.

In patients with suboptimal BP control despite antihypertensive therapy, assessment of medication adherence is essential. Hypertension is frequently asymptomatic, whereas antihypertensive medications may be associated with adverse effects that influence adherence. A clear discussion regarding the long-term risks of uncontrolled hypertension, along with counselling about potential medication side effects, may improve compliance.

Lifestyle modification remains a cornerstone of management and is often under-emphasised. NICE advises regular exercise, weight reduction, dietary modification (eg DASH diet), smoking cessation, reduction of alcohol and caffeine intake, and salt restriction.^4^, ^7^ Concomitant medications that adversely affect BP should also be reviewed and minimised where possible. The British and Irish Hypertension Society, Blood Pressure UK, and the British Heart Foundation are all excellent resources to support professionals and patients (see Resources, below).

## When to refer

Referral may be indicated by the clinical context of presentation, abnormal investigation findings, or the presence of hypertension in specific patient groups. Access to accredited specialist services and investigations is unevenly distributed across the UK, creating inequalities in access.^2^ In the absence of a dedicated hypertension clinic, patients are often referred to a local cardiologist. However, it is worth noting that not all cardiologists have specialist training or a clinical focus on hypertension, and some may have less overall experience in this than the referring clinician.^8^

While specialist input may be sought for any diagnostic or management challenges in hypertension, specific guideline-based referral indications should be considered, as summarised in Table 3. All referrals should be supported by a comprehensive clinical examination (Box 1), include results of baseline investigations for target organ damage (Table 3), duration of hypertension, age at diagnosis, antihypertensive treatment history, general medical history and family cardiovascular history. All patients with hypertension should undergo baseline investigations, including electrocardiography to assess for left ventricular hypertrophy, urinalysis, renal function testing, glycated haemoglobin (HbA1c), lipid profile, thyroid function tests, and measurement of the urine albumin–creatinine ratio.

**Table 3:** Guideline indications for referral to a hypertension specialist.

Presentation	Definition	Action	Guideline
***General adult population***			
Clinic BP ≥180/120 mmHg with symptoms^a^	Severe hypertension	Same-day specialist assessment	NICE CG136^4^
Clinic BP ≥180/120 mmHg without symptoms	Severe hypertension	Investigate for target organ damage (TOD) as soon as possible^b^ • If TOD identified, start immediate antihypertensive treatment • If no TOD identified, confirm diagnosis by repeating BP measurement within 7 days, considering ABPM or HBPM, and ensuring clinical review within 7 days^c^	NICE CG136^4^
Diagnosed hypertension under age 40	Young hypertension	Refer for specialist investigation for secondary causes, detailed assessment of lifetime cardiovascular risks, and review of the long-term balance of treatment benefit and risks	NICE CG 136^4^
Postural hypotension with symptoms	Fall in BP ≥20/10 mmHg after standing for at least 1 min	If symptoms of PH but BP falls <20/10 mmHg on standing from sitting, repeat on standing after 1 min from lying • If PH confirmed, address likely causes and review medications • Adopt standing BP for future management • If PH symptoms persist without postural drop, refer to specialist.	NICE CG136^4^
BP not controlled on three or more antihypertensives	Resistant hypertension	• Confirm BP above target with ABPM/HBPM • Assess for PH before further prescribing • Review medication adherence Following the above, add step 4 treatment as spironolactone if serum K+ ≤4.5 mmol/L, or with an α or β-blocker if K+ >4.5 mmol/L. Monitor renal function 1 month after starting. Referral for specialist investigation and management is indicated before or after initiating step 4.	NICE CG136^4^
***Women planning pregnancy or currently pregnant***			
Planning to conceive, or pregnant, with pre-existing hypertension	Chronic hypertension	Offer women with chronic hypertension referral to a specialist in hypertensive disorders of pregnancy to discuss the risks and benefits of treatment.	NICE NG133^9^
New onset of hypertension ≥140/90 mmHg during pregnancy	Gestational hypertension	A full assessment should be carried out in a secondary care setting by a healthcare professional who is trained in the management of hypertensive disorders of pregnancy.	NICE NG133
Hypertension ≥160/110 mmHg during pregnancy	Severe gestational hypertension	Admit to hospital, but if BP falls below 160/110 mmHg, then manage as for Hypertension	NICE NG133
	Pre-eclampsia & Eclampsia	Carry out a full assessment at each appointment; Admit if any concerns or: • sustained systolic BP ≥160 mmHg • new and persistent rise in creatinine (≥90 μmol/L • rise in alanine transaminase (≥over 70 IU/L or twice upper limit normal range • fall in platelet count (<150,000/μl) • signs of impending eclampsia, worsening pre-eclampsia or impending pulmonary oedema • suspected fetal compromise	NICE NG133
***Adults referred for elective surgery***			
	Pre-operative assessment	• There should be a record of BP taken in primary or secondary care <160/100 mmHg (or ABPM or HBPM <155/95 mmHg) within the previous 12 months. If not documented, BP should be measured • If clinic BP is ≥ 180/120 mmHg (or ABPM/HBPM ≥175/115 mmHg should not proceed to surgery, but be referred (usually back to their GP) to control BP.	AA/BIHS 2026^10^
**Footnotes**			

BP, blood pressure; TOD, target organ damage; NICE, National Institute for Health and Care Excellence; ABPM, ambulatory BP measurement; HBPM, home BP measurement; PH, postural hypotension; GP, general practitioner.

a Symptoms or signs indicating same-day referral are retinal haemorrhage, papilloedema, new onset of confusion, chest pain, heart failure, or acute kidney injury. Also applies if phaeochromocytoma suspected (e.g., from labile or postural hypotension, headache, palpitations, pallor, abdominal pain or diaphoresis).

b TOD is defined as damage to the heart, brain, kidneys or eyes. Consequently, investigations should include fundoscopy (for retinopathy), 12-lead ECG (to detect LVH), urinalysis for haematuria and albumin: creatinine ratio, and blood analysis for glycated haemoglobin, electrolytes, creatinine, estimated glomerular filtration rate, total cholesterol and HDL cholesterol (for estimation of cardiovascular risk). These investigations are the minimum indicated for all people suspected to have hypertension, and results of such should be included in any referral.

c Clinical review could be by GP, especially if newly diagnosed, or by specialist if other table criteria apply.

Box 1 **Clinical points to cover in assessing hypertension.**

Eyes: fundoscopy for retinopathy

Neurological: evidence of cerebrovascular disease and cognitive impairment

Vascular: unequal BP, evidence of peripheral vascular disease

Pulse palpation: arrhythmia, peripheral pulses present, radio-femoral delay

Auscultation for carotid, femoral, and renal artery bruits; signs of peripheral arterial disease

Cardiac auscultation: valvular heart disease and cardiomegaly, signs of heart failure

Abdominal: palpable kidneys (polycystic kidney disease or mass) and aortic enlargement

General: e.g., cushingoid features or thyroid disease

## Referral criteria in the general population

For the general adult population, NICE sets out and periodically updates clinical guidance on the management of hypertension, including indications for referral (Table 3).^4^ Same-day specialist referral is required when BP ≥180/120 mmHg with symptoms of hypertensive emergency.^11^, ^12^ Most UK centres do not have same-day specialist hypertension clinics, hence these patients may need to be referred to same-day emergency care or the emergency department.

Routine referral should be considered for adults aged <40 years with hypertension. The most recent NICE guideline update (November 2023) recognised that postural hypotension is generally poorly assessed, but may require referral to a specialist if persistent.^13^ Although no longer a NICE referral recommendation, a systolic inter-arm BP difference ≥15 mmHg or lower is associated with additional cardiovascular risk, and should be taken into consideration.^4^, ^14^

If blood pressure remains above target (≤140/90 mmHg, or ≤150/90 mmHg in adults aged ≥80 years) despite treatment with maximally tolerated doses of an angiotensin converting enzyme-inhibitor (ACE-I) or angiotensin receptor blocker (ARB), a calcium channel blocker and a thiazide-like diuretic, specialist referral should be considered for a diagnosis of resistant hypertension, before or after escalation to step 4 therapy. In patients with stable renal function (and potassium level <4.5 mmol/l), step 4 treatment typically involves adding spironolactone.^15^ If renal function is not favourable, consider an alpha-blocker (eg doxazosin) or a beta-blocker as alternatives.

Specialist assessment may include repeat ABPM to confirm true treatment resistance and identify pseudo-resistant hypertension due to poor adherence, optimisation and escalation of therapy, investigation for secondary hypertension, and consideration of cost-effective non-pharmacological interventions such as renal denervation.^16^

## Screening for secondary hypertension: when and how

Primary (essential) hypertension is the most common form of hypertension. It lacks a specific, identifiable, underlying cause amenable to intervention. Its pathophysiology is not fully understood, but is considered to result from a complex interplay of genetic predisposition, environmental influences and age-related vascular stiffening.^17^ In contrast, secondary hypertension has an identifiable underlying cause or precipitating factor. It accounts for 5–10% of cases and may be suggested by abnormal findings on assessment for target organ damage (Table 3) or by specific clinical features, including characteristic symptoms, signs or demographic factors (Box 2). Screening for secondary causes of hypertension should be guided by the clinical history, examination and baseline assessment for target organ damage.

Box 2 **Indications to screen for secondary causes of hypertension.**

• Resistant hypertension
• Early onset hypertension (age <40)
• Sudden onset or rapidly worsening hypertension
• Severe hypertension (≥180/120 mmHg)
• Hypokalaemia on initial renal function testing
• TOD out of proportion for the degree or duration of hypertension
• Specific clinical clues, such as episodic headache and palpitations, renal asymmetry, abdominal bruits, or features of endocrine disease

Primary aldosteronism (PA) is the most common endocrine cause of secondary hypertension (prevalence of 2–6% among hypertensives). Screening should be considered in adults <40 years, in the presence of spontaneous or thiazide-induced hypokalaemia (serum potassium <3.5 mmol/L), resistant hypertension, family history or adrenal incidentalomas detected on imaging. However, absence of hypokalaemia or adrenal adenomas do not exclude PA and screening should be still considered if clinically suspected.^11^

Screening involves measurement of the plasma aldosterone–renin ratio (ARR). Hypokalaemia should be corrected before testing. Ideally testing should be done after anti-hypertensive washout; however, this may not always be practicable or routinely required, unless there is a concern that results may be false positive or negative, or in patients with moderate–high pre-test probability.^18^ Confirmation requires dynamic testing, such as oral sodium loading test, saline infusion test, captopril challenge test, or fludrocortisone suppression test, depending on local expertise. Adrenal imaging and adrenal vein sampling are then used to identify candidates for unilateral adrenalectomy.^19^

If PA is suspected, consider deferring initiation of renin–angiotensin–aldosterone system (RAAS) influencing agents until the ARR has been measured.

Investigation for renal causes should be guided by clinical findings (eg renal mass or bruit), abnormal renal function or urinalysis. Renal ultrasound may inform the need for further imaging and specialist assessment. Renal artery stenosis can be associated with resistant and/or young-age onset hypertension, especially in women below the age of 50. Consider dedicated imaging (Doppler, CT or MRI) in these patients, especially if there is associated renal dysfunction (particularly with ACE-I or ARB treatment), small renal size or history of fibromuscular dysplasia. Plasma free metanephrines or urinary fractionated metanephrines measurement is indicated to exclude phaeochromocytoma or paraganglioma, particularly in patients with adrenal mass, paroxysmal hypertension, episodic headache, sweating or palpitations.^20^

Obstructive sleep apnoea (OSA) is a leading yet underdiagnosed cause of secondary hypertension. It causes nocturnal BP spikes and sustained daytime hypertension due to sympathetic nerve activation from interrupted breathing. Screening using the Epworth score and sleep studies can make the diagnosis. Treatment with CPAP (continuous positive airway pressure) therapy can reduce BP, though antihypertensive medication is often needed.^21^

## Referral in special circumstances

### Pregnancy and conception

Hypertensive disorders affect 8–10% of pregnancies and are associated with significant maternal and fetal morbidity. Hypertension may be present prior to pregnancy or diagnosed before 20 weeks’ gestation (chronic hypertension), present as new-onset hypertension after 20 weeks’ gestation (gestational hypertension), or occur as new-onset hypertension with multi-organ involvement (pre-eclampsia).

The NICE guideline *Hypertension in pregnancy: diagnosis and management* (NG133) aims to reduce the associated maternal and fetal risks and outlines referral criteria (summarised in Table 3).^9^ Suspected pre-eclampsia or severe hypertension requires same-day specialist referral.^11^ In women with pre-existing hypertension, pre-pregnancy or early pregnancy specialist assessment is recommended to optimise management and reduce the risks associated with inadequate blood pressure control. Additionally, women who develop gestational hypertension require specialist investigation to identify complications, including pre-eclampsia.

Antihypertensive therapy should be reviewed promptly once pregnancy is confirmed, and medications contraindicated in pregnancy or breastfeeding should be replaced with safer alternatives. It should not be forgotten that gestational hypertension can lead to chronic hypertension in later life, so long-term follow-up and lifestyle advice are warranted.^22^

### Pre-operative assessment

Pre-operative BP assessment can be challenging due to spuriously elevated readings related to white-coat and environmental effects. To minimise unnecessary referrals, the Association of Anaesthetists and the British and Irish Hypertension Society (AA/BIHS) developed guidance on pre-operative BP assessment. Updated in 2026, this prioritises recorded primary care BP values over clinic readings and supports proceeding with routine surgery without intervention, provided that BP does not exceed 180/120 mmHg (Table 3).^10^

## Summary

Most patients with hypertension do not require referral to specialist services. However, clinical history, presentation and initial investigations provide important indicators for patients who may benefit from specialist review. Effective and comprehensive management of hypertension requires awareness of referral criteria and collaborative care from generalists and specialists.

## Useful resources

### British Heart Foundation

For a wealth of lifestyle and other information, for patients and professionals, see: https://www.bhf.org.uk/.

### British and Irish Hypertension Society

For professional guidance and statements on all aspects of hypertension, see: https://bihs.org.uk/.

### Blood Pressure UK

UK’s patient-focused BP charity with resources and advice, see: https://www.bloodpressureuk.org/.

### NICE Guidance

https://www.nice.org.uk/guidance/ng136.

### NICE visual summaries

#### Adult hypertension

https://www.nice.org.uk/guidance/ng136/resources/visual-summary-pdf−6899919517; accessed 2/2/26.

#### Hypertension in pregnancy

https://www.nice.org.uk/guidance/ng133/resources/gestational-hypertension-antenatal-care-pdf−8720711394; accessed 2/2/26.

## CRediT authorship contribution statement

**Christopher E. Clark:** Writing – review & editing, Writing – original draft, Visualization, Supervision, Resources, Conceptualization. **Anish George:** Writing – review & editing, Resources.

## Funding

CEC is supported by the National Institute for Health and Care Research (10.13039/100006662NIHR) School for Primary Care Research (SPCR) and the Southwest GP Trust (SWGPT).

AG has no funding declarations.

The views expressed are those of the authors and not necessarily those of the NIHR, SPCR, SWGPT, BIHS, NICE or the Department of Health and Social Care.

## Declaration of competing interest

The authors declare the following financial interests/personal relationships which may be considered as potential competing interests: Christopher Clark reports financial support was provided by University of Exeter Medical School. CEC is supported by the National Institute for Health and Care Research (NIHR) School for Primary Care Research (SPCR) and the Southwest GP Trust (SWGPT). He chairs the BIHS Blood Pressure Technology Committee and is a NICE hypertension guideline committee member. The views expressed are those of the authors and not necessarily those of the NIHR, SPCR, SWGPT, BIHS, NICE or the Department of Health and Social Care. AG has no declarations. If there are other authors, they declare that they have no known competing financial interests or personal relationships that could have appeared to influence the work reported in this paper.
